# Prevention of Infection with *Mycobacterium tuberculosis* by H4:IC31® Vaccination or BCG Revaccination in Adolescents 

**DOI:** 10.1056/NEJMoa1714021

**Published:** 2018-07-12

**Authors:** Elisa Nemes, Hennie Geldenhuys, Virginie Rozot, Kathryn Tucker Rutkowski, Frances Ratangee, Nicole Bilek, Simbarashe Mabwe, Lebohang Makhethe, Mzwandile Erasmus, Asma Toefy, Humphrey Mulenga, Willem A. Hanekom, Steven G. Self, Linda-Gail Bekker, Robert Ryall, Sanjay Gurunathan, Carlos A. DiazGranados, Peter Andersen, Ingrid Kromann, Thomas Evans, Ruth D. Ellis, Bernard Landry, David A. Hokey, Robert Hopkins, Ann M. Ginsberg, Thomas J. Scriba, Mark Hatherill

**Affiliations:** South African Tuberculosis Vaccine Initiative, Institute of Infectious Disease & Molecular Medicine and Division of Immunology, Department of Pathology, University of Cape Town, South Africa; South African Tuberculosis Vaccine Initiative, Institute of Infectious Disease & Molecular Medicine and Division of Immunology, Department of Pathology, University of Cape Town, South Africa; South African Tuberculosis Vaccine Initiative, Institute of Infectious Disease & Molecular Medicine and Division of Immunology, Department of Pathology, University of Cape Town, South Africa; Aeras, Rockville, Maryland, USA; South African Tuberculosis Vaccine Initiative, Institute of Infectious Disease & Molecular Medicine and Division of Immunology, Department of Pathology, University of Cape Town, South Africa; South African Tuberculosis Vaccine Initiative, Institute of Infectious Disease & Molecular Medicine and Division of Immunology, Department of Pathology, University of Cape Town, South Africa; South African Tuberculosis Vaccine Initiative, Institute of Infectious Disease & Molecular Medicine and Division of Immunology, Department of Pathology, University of Cape Town, South Africa; South African Tuberculosis Vaccine Initiative, Institute of Infectious Disease & Molecular Medicine and Division of Immunology, Department of Pathology, University of Cape Town, South Africa; South African Tuberculosis Vaccine Initiative, Institute of Infectious Disease & Molecular Medicine and Division of Immunology, Department of Pathology, University of Cape Town, South Africa; South African Tuberculosis Vaccine Initiative, Institute of Infectious Disease & Molecular Medicine and Division of Immunology, Department of Pathology, University of Cape Town, South Africa; South African Tuberculosis Vaccine Initiative, Institute of Infectious Disease & Molecular Medicine and Division of Immunology, Department of Pathology, University of Cape Town, South Africa; South African Tuberculosis Vaccine Initiative, Institute of Infectious Disease & Molecular Medicine and Division of Immunology, Department of Pathology, University of Cape Town, South Africa; Statistical Center for HIV Research, Vaccine and Infectious Disease Division, Fred Hutchinson Cancer Research Center, Seattle, Washington, USA; The Desmond Tutu HIV Centre, University of Cape Town, Cape Town, South Africa; Sanofi Pasteur, Swiftwater, PA, USA; Sanofi Pasteur, Swiftwater, PA, USA; Sanofi Pasteur, Swiftwater, PA, USA; Statens Serum Institut, Copenhagen, Denmark; Statens Serum Institut, Copenhagen, Denmark; Aeras, Rockville, Maryland, USA; Aeras, Rockville, Maryland, USA; Aeras, Rockville, Maryland, USA; Aeras, Rockville, Maryland, USA; Aeras, Rockville, Maryland, USA; Aeras, Rockville, Maryland, USA; South African Tuberculosis Vaccine Initiative, Institute of Infectious Disease & Molecular Medicine and Division of Immunology, Department of Pathology, University of Cape Town, South Africa; South African Tuberculosis Vaccine Initiative, Institute of Infectious Disease & Molecular Medicine and Division of Immunology, Department of Pathology, University of Cape Town, South Africa

## Abstract

**Background:**

Recent *Mycobacterium tuberculosis (M.tb*) infection predisposes to tuberculosis disease, the leading global infectious disease killer. We tested safety andefficacy of H4:IC31® vaccination or Bacille Calmette-Guerin (BCG) revaccination for prevention of *M.tb* infection.

**Methods:**

QuantiFERON-TB Gold In-tube (QFT) negative, HIV-uninfected, remotely BCG-vaccinated adolescents were randomized 1:1:1 to placebo, H4:IC31® or BCG revaccination (NCT02075203). Primary outcomes were safety and acquisition of *M.tb* infection, defined by initial QFT conversion tested 6-monthly over two years. Secondary outcomes were immunogenicity and sustained *M.tb* infection, defined by sustained QFT conversion without reversion three and six months post-conversion. Statistical significance for efficacy proof-of-concept was set at 1-sided p<0.10.

**Results:**

990 participants were enrolled. Both vaccines had acceptable safety profiles and were immunogenic. QFT conversion occurred in 134 and sustained conversion in 82 participants. Neither H4:IC31® nor BCG prevented initial QFT conversion, with efficacy point estimates of 9.4% (95% confidence interval: -36.2, 39.7; one-sided p=0.32) and 20.1% (-21.0, 47.2; one-sided p=0.14), respectively. However, BCG did prevent sustained QFT conversion with an efficacy of 45.4% (6.4, 68.1; one-sided p=0.013); H4:IC31® efficacy was 30.5% (-15.8, 58.3; one-sided p=0.08). QFT reversion rate from positive to negative was 46% in BCG, 40% in H4:IC31 and 25% in placebo recipients.

**Conclusions:**

This first proof-of-concept, prevention of *M.tb* infection trial showed that sustained infection can be prevented by vaccination in a high-transmission setting and confirmed feasibility of this strategy to inform clinical development of new vaccine candidates. Evaluation of BCG revaccination to prevent tuberculosis disease in *M.tb*- uninfected populations is warranted.

## Introduction

*Mycobacterium tuberculosis (M.tb)* causes more deaths worldwide than any other infectious agent and is increasingly characterized by antimicrobial resistance^1^. New preventative tools are essential to end the tuberculosis (TB) epidemic^1^. Vaccines that prevent pulmonary TB in adolescents and young adults would have major impact on control of drug-sensitive and multidrug-resistant TB by interrupting transmission^2^.

Development of new TB vaccines is hampered by lack of validated preclinical models and human immune correlates of protection to provide evidence for advancing candidates into late-stage trials. *M.tb* exposure may result in early elimination of bacteria by innate or adaptive immunity; or establishment of infection, which may remain asymptomatic (latent) in most individuals or progress to active disease^3^. Vaccine-mediated prevention of *M.tb* infection (POI) could be an important signal of efficacy against TB disease. Further, size and duration of a POI trial are less than for a trial of disease prevention, since *M.tb* infection occurs more frequently^4-6^.

Acquisition, persistence and clearance of asymptomatic M.tb infection cannot be measured directly. Diagnosis of *M.tb* infection is based on immunological sensitization to *M.tb* antigens, assessed by tuberculin skin test (TST), which cross-reacts with other mycobacteria including Bacille Calmette-Guerin (BCG) vaccine. IFNγ release assays, including the QuantiFERON- TB Gold In-tube assay (QFT), are more specific for *M.tb*, but may yield false positive/negative results due to assay variability and uncertainty around the optimal assay cut-off^7,8^. Although neither TST nor QFT distinguishes between infection and disease, recent infection, diagnosed by TST or QFT conversion from negative to positive, is associated with increased disease risk, compared to non-conversion or remote conversion^8-11^. Human and animal studies suggest TST reversion is associated with early containment of *M.tb* infection and lower risk of TB disease^4,12-14^, perhaps indicating sterilization. QFT can also revert from positive to negative^11^. Although the clinical significance of QFT reversion remains to be established^11^, we propose that sustained QFT conversion is more likely associated with sustained *M.tb*b infection and progression to disease than transient QFT conversion. These observations suggest that vaccine-mediated prevention of initial or sustained *M.tb* infection could be critical steps towards TB control.

 Observational studies indicate that primary BCG vaccination may offer partial protection against *M.tb* infection^15-18^. BCG revaccination may also protect against sustained *M.tb* infection, but this hypothesis has not previously been tested in a prospective, randomized, placebo-controlled trial^19^. Two large randomized trials showed no benefit of BCG revaccination for protection against TB disease^20-22^, but neither trial enrolled based on *M.tb* infection status or measured infection acquisition during follow-up. H4:IC31® is a candidate subunit vaccine, consisting of mycobacterial antigens Ag85B and TB10.4, which do not cross-react with QFT, together with the IC31® adjuvant (see Supplementary Appendix). H4:IC31® has shown protection in pre-clinical models^23-25^ and acceptable safety and immunogenicity in humans^26,27^.

 Demonstration of efficacy in a POI trial would provide strong impetus for larger trials to test H4:IC31® or BCG revaccination efficacy in preventing TB disease in *M.tb*-uninfected populations, allow identification of immune correlates of vaccine-mediated protection, and confirm the utility of the POI trial design to identify promising TB vaccine candidates. This clinical trial evaluated safety, immunogenicity, and prevention of initial and sustained QFT conversion by H4:IC31® or BCG revaccination in healthy South African adolescents in a high TB transmission setting^11^. 

### Methods

#### Trial design

This phase II, randomized, three-arm, placebo-controlled, partially-blinded clinical trial aimed to enroll 990 healthy, HIV-uninfected, QFT-negative, 12- to 17-year-old adolescents, BCG- vaccinated in infancy, at two South African sites (Table 1). Adolescents with previously treated or current TB, a household TB contact, substance use, or pregnancy were excluded. Adolescents provided written informed assent and parents/legal guardians written informed consent. Regulatory approvals, consent procedures and inclusion/exclusion criteria are detailed in the Supplementary Appendix. 

**Table 1:** Participant baseline characteristics (safety population)

Variable		Statistic	Placebo (n=329)	H4:IC31® (n=330)	BCG (n=330)	Total (n=989)
Site	SATVI	n (%)	306 (93.0)	306 (92.7)	305 (92.4)	917 (92.7)
	Emavundleni	n (%)	23 (7.0)	24 (7.3)	25 (7.6)	72 (7.3)
Age (years)1		Median (min, max)	14 (12, 17)	14 (12, 17)	14 (12, 17)	14 (12, 17)
Self-declared Race1	Asian n (%)	1 (0.3)	1 (0.3)	1 (0.3)	3 (0.3)
	Black African n (%)	120 (36.5)	120 (36.4)	126 (38.2)	366 (37.0)
	Caucasian n (%)	1 (0.3)	1 (0.3)	3 (0.9)	5 (0.5)
	Cape Mixed Ancestry n (%)	207 (62.9)	208 (63.0)	200 (60.6)	615 (62.2)
Sex (females)1		n (%)	169 (51.4)	189 (57.3)	162 (49.1)	520 (52.6)
Body mass index (kg/m2)1		Median (min, max)	19.9 (14.3, 36.8)	19.6 (13.8, 38.3)	19.4 (13.1, 36.9)	19.6 (13.1, 38.3)

1 Numbers are presented for both sites combined

Eligible participants were enrolled into two sequential cohorts, each randomized 1:1:1 to receive intramuscular saline placebo or H4:IC31® (15μg H4 polyprotein, Sanofi Pasteur, and 500nmol IC31®, Statens Serum Institut) on Day 0 (D0) and D56, or intradermal BCG (2- 8x105 CFU, Statens Serum Institut) at D0. 

**Figure 1: Study design and CONSORT diagram:**
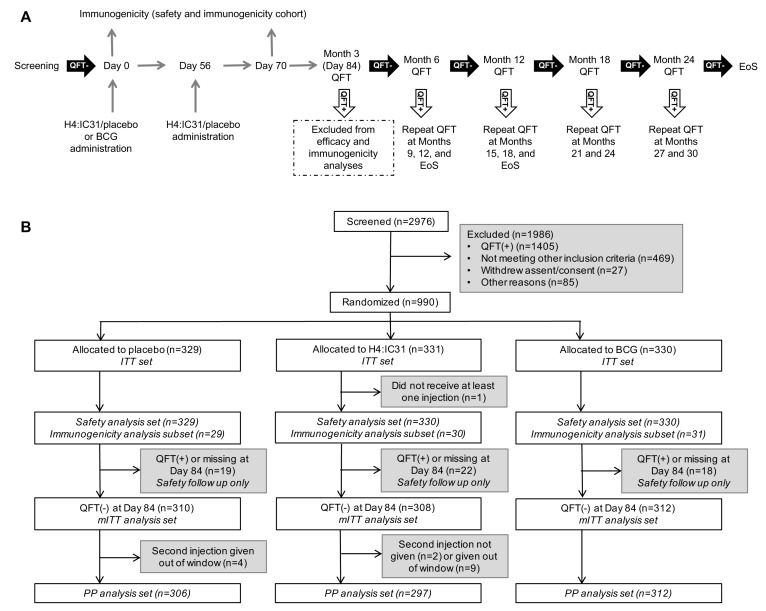
(A) Study design. Each participant followed a schedule of evaluations according to study arm and QFT test results. An 84-day wash-out period was implemented to account for participants who may already have been M.tb-infected at enrollment but had not yet QFT converted. After the primary analysis, the IDMC recommended that participants who converted at Month 6 or 12 should return for an additional end-of-study visit to evaluate sustained QFT conversion. Safety outcomes were assessed at each study visit, including evaluation of symptoms of TB disease. QFT, QuantiFERON-TB Gold In-tube; EoS, end of study. CONSORT diagram. Amongst screened individuals (n=2976), 1986 were excluded for one or more reasons. The most common reason for ineligibility was a positive QFT test (n=1405, 71%); other common reasons for exclusion were: abnormal blood results (n=244, 12%), body mass index out of range (n=122, 6%), previous TB or household TB contact (n=55, 3%). ITT, intent-to-treat; mITT, modified ITT; PP, per protocol.

In the first cohort of 90 participants, approximately 30 per arm, additional safety tests and immunogenicity assays were performed (Supplementary Appendix). The follow-up schedule of individual participants was contingent on QFT results at D84 and Months 6, 12, 18 and 24 (Figure 1A). An 84-day ‘wash-out’ period was stipulated to exclude participants who may have been *M.tb*-infected at baseline, but not yet QFT-positive. Participants who tested QFT- positive at D84 were followed for 6 months after last vaccination for safety but excluded from efficacy evaluations (Figure 1B). An Independent Data Monitoring Committee (IDMC) reviewed D7 and D84 safety data from the first cohort and safety and efficacy data for all participants throughout follow-up (Supplementary Appendix). 

South African guidelines do not recommend preventive antimicrobials for HIV-negative, *M.tb*-infected persons >5 years old; therefore therapy was not provided to converters28. 

#### Safety Outcomes

Solicited adverse events (AEs) were recorded for 7 days, unsolicited AEs for 28 days and injection site AEs for 28 days after placebo and H4:IC31®, or 84 days after BCG. Serious adverse events (SAEs) and adverse events of special interest (AESIs) were recorded for the entire study period (Supplementary Appendix). 

#### Immunogenicity Outcomes 

Immunogenicity was evaluated by intracellular cytokine staining (ICS)29 and flow cytometry (Supplementary Appendix; Table S1). 

#### Efficacy Outcomes 

We prioritized efficacy assessment using the modified intent-to-treat (mITT) population, defined as those who received at least one injection and had not converted to QFT-positive at D84. 

The primary efficacy endpoint was initial QFT conversion using the threshold of IFNγ ≥0.35 International Units (IU)/mL at any time after D84 and was compared for the H4:IC31® or BCG revaccination arms *versus* placebo. 

The QFT assay was conducted according to the manufacturer’s instructions, with additional, more stringent parameters to reduce variability and improve reproducibility^8^ (Supplementary Appendix). 

 The secondary efficacy endpoint was sustained QFT conversion without reversion through 6 months after initial QFT conversion, *i.e.*, three consecutive positive QFT results after D84 (Figure 1A). 

In this study, QFT conversion and sustained QFT conversion were considered surrogate endpoints for *M.tb* infection and sustained *M.tb* infection, respectively. 

Exploratory efficacy endpoints included evaluation of sustained conversion through end of study (EoS) and alternative QFT threshold values for initial and sustained conversion, including IFNγ <0.2IU/mL to >0.7IU/mL8, and IFNγ <0.35IU/mL to >4IU/mL30, as detailed in 

#### Randomization and blinding

Group allocation was concealed by an interactive web response system. Assignment was based on block randomization to placebo, H4:IC31® or BCG (1:1:1), stratified by school (Worcester site) or residential area (Emavundleni site). 

Blinding was partial because BCG causes a recognizable injection site reaction and is administered once. However, randomization to H4:IC31® and placebo was double-blinded: syringe contents were masked, injection volumes were identical, and injections were administered by a research nurse who did not perform post-enrollment study procedures or data collection. Laboratory personnel were blinded to all three treatment groups. 

Primary and secondary efficacy endpoints were analyzed using two log-rank statistics (H4:IC31® or BCG versus placebo, α=0.1, one-sided) without adjustment for multiplicity. Efficacy estimates were based on hazard ratios from a Cox regression model. Analyses and endpoints are detailed in the Supplementary Appendix. All other analyses were two-sided (α=0.05). 

### Results

#### Baseline characteristics

Between 1 April 2014 and 25 May 2015, 2,976 participants were screened and 990 were enrolled. Most (1405/1986, 71%) exclusions were due to positive QFT (Figure 1). Baseline characteristics did not differ among arms (Table 1). The final visit occurred on 28 August 2017). Loss to follow-up was 4% (41/990) through EoS (Figure 1). 

#### Safety

Safety was assessed in all participants who received at least one injection. Each vaccine had an acceptable safety profile (Table S2); 550 participants experienced at least one AE. H4:IC31® and placebo had similar AE profiles. AEs were more frequent in the BCG arm; 98.8% experienced at least one event. These were predominantly local injection site AEs of mild-to-moderate severity, consistent with BCG’s known reactogenicity profile31. Upper respiratory tract infections occurred less frequently in the BCG arm compared to placebo and H4:IC31 arms (2.1%, 7.9%, and 9.4%, respectively; p<0.001). In total, 4 severe AEs, 19 SAEs, and no AESIs or severe related AEs were observed. There was no clinically significant difference in the rate of severe AEs or SAEs between study arms. One participant in the placebo arm died from suicide. 

#### Immunogenicity

**Figure 2: Immunogenicity:**
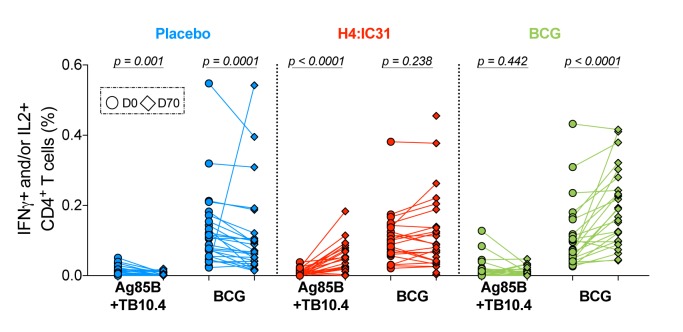
Vaccine immunogenicity measured by PBMC intracellular cytokine staining (ICS) and flow cytometry following stimulation with Ag85B or TB10.4 peptide pools (summed response is shown) or BCG. Paired responses of CD4 T cells expressing IFNγ and/or IL2 for each individual (between 23 and 28 participants were included at each time point) at D0 (circles) and D70 (diamonds) randomized to placebo (blue), H4:IC31® (red) or BCG (green). Changes in response between D0 and D70 were compared by Wilcoxon Signed-Rank Test.

Frequencies of cytokine-expressing antigen-specific T cells were assessed at baseline and D70 by ICS (Figure 2). Ag85B- and TB10.4-specific CD4 T cell responses were low before vaccination and H4:IC31® induced significant increases in these responses. By contrast, high levels of pre-vaccination BCG-specific CD4 T cell responses were observed in all arms. BCG revaccination boosted the BCG-specific CD4 T cell responses significantly (Figure 2 and Figure S1). 

#### Efficacy

**Figure 3: Vaccine efficacy:**
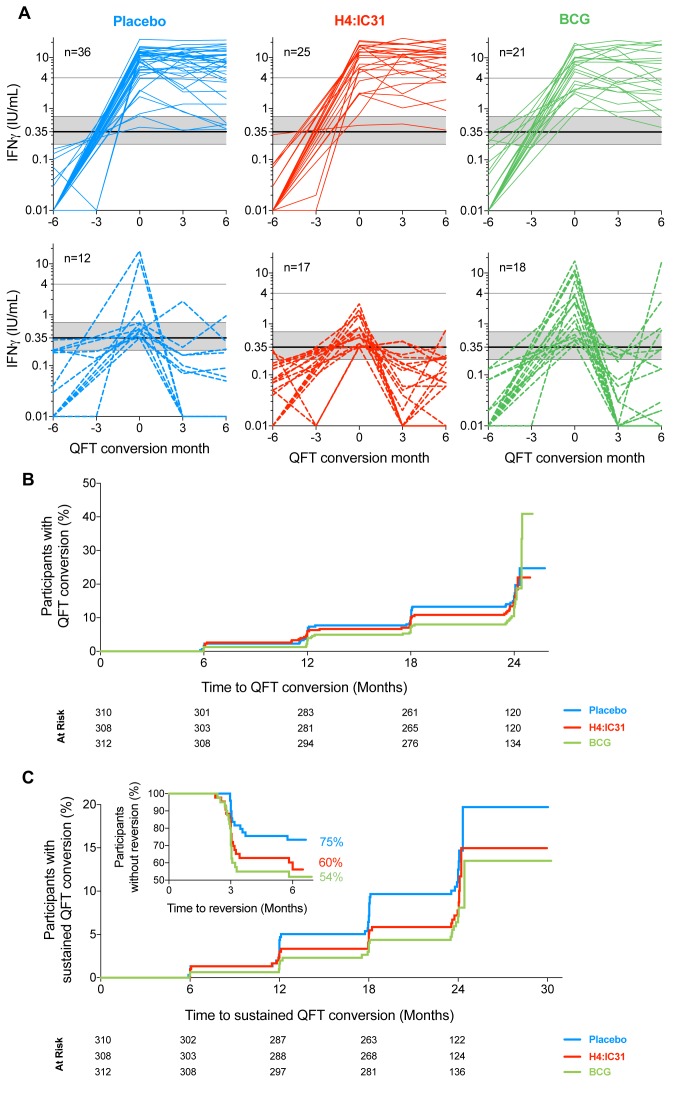
(A) Longitudinal quantitative IFNγ values measured by QFT by study arm, aligned to initial QFT conversion time point (month 0). Each line represents one individual; those who never converted and those with missing QFT results after initial conversion are not shown. Solid lines denote participants who met the secondary efficacy endpoint (sustained QFT conversion, top row) and dashed lines denote participants with initial QFT conversion who then reverted (bottom row). The solid horizontal line denotes the manufacturer’s recommended threshold (0.35IU/mL); the shaded horizontal area denotes the uncertainty zone (0.2-0.7IU/mL); the horizontal line at 4.0IU/mL denotes an alternative QFT threshold applied in exploratory analyses. Values <0.01IU/mL were set to 0.01 to enable plotting on the log scale. (B) Kaplan-Meier curves representing time to initial QFT conversion (primary efficacy endpoint) after first vaccination by study arm in the mITT population. Statistics are reported in Table 2. (C) Kaplan-Meier curves representing time after first vaccination to initial QFT conversion in participants with sustained conversion (secondary efficacy endpoint) by study arm in the mITT population. Inset depicts time to QFT reversion within 6 months of initial conversion in participants with QFT values at three and six months post-conversion. Statistics for conversion endpoints are reported in Table 2.

Sixty (6.1%) participants were excluded from the mITT population (Figure 1). There were 134 initial QFT conversions (14.4% or 9.9/100 person-years; Figure S2A) in the mITT population, with a high QFT reversion rate (50 of 133 with repeated QFT, 37.6%). There were 82 sustained converters (8.8% of all participants; 62.6% of initial converters with non- missing QFT results; Figure 3A). Median time to initial QFT conversion among converters was 15.0 months. No TB disease cases were identified. 

Neither H4:IC31® vaccination nor BCG revaccination met the primary efficacy criterion, based on initial QFT conversion rates (Table 2; Figure 3B). However, H4:IC31® efficacy point estimate for prevention of sustained QFT conversion, the secondary endpoint, was 30.5% (one-sided p=0.08; 95% CI: -15.8, 58.3%; Table 2; Figure 3C), with 17/43 (39.5%) reversions among converters with non-missing QFT data. H4:IC31® efficacy for prevention of sustained QFT conversion at EoS was 34.2% (one-sided p=0.05; 95% CI: -10.4, 60.7%; Table 2). 

**Table 2:** Vaccine efficacy

Arms		Placebo	H4:IC31®					BCG				
Endpoint	QFT conversion threshold	n/N (%)	n/N (%)	Vaccine efficacy				n/N (%)	Vaccine efficacy			
				Point Est (%)	80% CI	95% CI	p-val		Point Est (%)	80% CI	95% CI	p-val
**Primary endpoint**												
QFT conversion1	≥ 0.35IU/mL	49/310 (15.8)	44/308 (14.3)	**9.4**^2^	-18.3, 30.6	-36.2, 39.7	0.32^3^	41/312 (13.1)	20.1^2^	-4.8, 39.1	-21.0, 47.2	0.14^3^
**Secondary endpoint**												
Sustained QFT conversion4	≥ 0.35IU/mL	36/310 (11.6)	25/308 (8.1)	**30.5**^2^	3.0, 50.2	-15.8, 58.3	0.08^3^	21/312 (6.7)	45.4^2^	22.3, 61.6	6.4, 68.1	0.01^3^
**Exploratory endpoint**												
Sustained QFT conversion5	<0.2 to >0.7IU/mL	31/310 (10.0)	24/308 (7.8)	**23.2**^2^	-8.8, 45.8	-30.9, 54.9	0.16^3^	19/312 (6.1)	**41.6**^2^	15.2, 59.8	-3.3, 67.0	0.03^3^
End-of Study sustained QFT conversion6	≥ 0.35IU/mL	36/310 (11.6)	24/308 (7.8)	**34.2**^2^	7.7, 53.0	-10.4, 60.7	0.05^3^	20/312 (6.4)	**48.2**^2^	25.9, 63.8	10.5, 70.0	0.008^3^
QFT conversion7	> 4IU/mL	33/310 (10.6)	22/308 (7.1)	**34.5**^8^	6.8, 54.2	-12.1, 62.3	0.13^9^	19/312 (6.1)	**45.1**^8^	20.5, 62.2	3.8, 69.3	0.04^9^

1 QFT conversion from negative (< 0.35 IU/mL) at Day 84 to positive ( 2 ≥ 0.35 IU/mL) at any time point through end of study.

2 Vaccine efficacy point estimates and 80% CI and 95% CI are based on the hazard ratio estimated from the Cox regression model (two-sided).

3 P-values are based on a one-sided log-rank test compared to placebo. No multiplicity adjustment done for p-values.

4 QFT conversion from negative (< 0.35 IU/mL) at Day 84 to positive (≥ 0.35 IU/mL) at any time point through end of study, and without a change in QFT from positive to negative through 6 months after QFT conversion (excluding end-of-study call-back visit for participants who converted at Month 6 or 12).

5 QFT conversion from negative at Day 84 to positive at any time point through end of study, using an alternative threshold of < 0.2 IU/mL at any time point prior to conversion and > 0.7 IU/mL at conversion and maintained through 6 months after initial conversion (excluding end-of-study call-back visitfor participants who converted at Month 6 or 12).

6 QFT conversion from negative (< 0.35 IU/mL) at Day 84 to positive (≥ 0.35 IU/mL) at any time point through end of study, and without a change inQFT from positive to negative through 6 months after QFT conversion as well as the end-of-study call-back visit for participants who converted at Month 6 or 12.

7 QFT conversion from negative (< 0.35 IU/mL) at Day 84 to positive (> 4.0 IU/mL) at any time point through end of study.

8 Vaccine efficacy point estimates and 95% CI are calculated based on the conditional binomial procedure (Clopper-Pearson method with mid-p correction).

9 Two-sided p-values are based on Pearson Chi-square test.

Abbreviations: Point est = point estimate; CI = confidence interval; p-val = p-value

BCG revaccination efficacy for prevention of sustained QFT conversion was 45.4% (one- sided p=0.01; 95% CI: 6.4, 68.1%; Table 2; Figure 3C); 48.2% efficacy was observed at EoS (one-sided p=0.008; 95% CI: 10.5, 70.0%; Table 2). This BCG-induced effect was explained by a near-two-fold higher 6-month QFT reversion rate after conversion, compared to placebo recipients (19/41, 46.3% vs 12/49, 24.5%). 88% of all reversions occurred by 3 months post- conversion (Figure 3C inset). 

In exploratory analyses, efficacy of BCG revaccination for sustained QFT conversion was 41.6% using a stringent QFT conversion threshold (<0.2IU/mL to >0.7IU/mL) (one-sided p=0.03; 95% CI: -3.3, 67.0%); no significant effect of H4:IC31 was noted. BCG efficacy for initial conversion using the most stringent QFT threshold, >4.0IU/mL, was 45.1% (two-sided p=0.04; 95% CI: 3.8, 69.3%; Table 2; Figure S2B); no significant effect of H4:IC31 was noted at the 95% confidence level, although it did show significance at the less stringent confidence level (80% CI: 6.8, 54.2%). Additional exploratory analyses are reported in Table S3. 

Estimates of efficacy based on primary and secondary endpoints were not affected by sex, race or study site in *post-hoc* analysis (data not shown). 

### Discussion

We performed the first randomized controlled prevention of *M.tb* infection trial and showed that vaccination can reduce the rate of sustained *M.tb* infection in a high-transmission setting. 

Neither H4:IC31® nor BCG revaccination prevented initial QFT conversion. H4:IC31® showed 30.5% efficacy against sustained QFT conversion, which met the pre-defined significance threshold (one-sided p<0.1) as the first proof-of-concept efficacy signal ever observed for a subunit TB vaccine candidate. Although this modest effect did not meet stringent statistical criteria for demonstration of efficacy typically used in a licensure trial (95% CI), it provides an indication that subunit vaccines comprising few *M.tb* antigens may have biological effect and supports clinical evaluation of next-generation subunit vaccine candidates. 

BCG revaccination demonstrated 45.4% efficacy against sustained QFT conversion and met the more stringent statistical criterion. The durability of this important finding and potential public health significance for protection against TB disease warrants modeling and further clinical evaluation. We showed that vaccine-mediated protection against sustained QFT conversion may inform clinical development of vaccine candidates before entry into large- scale prevention of disease efficacy trials. Our findings, and availability of stored biospecimens, also provide an opportunity to discover immune responses that correlate with protection against infection, which would enable new TB vaccine design and evaluation. The efficacy signal for BCG revaccination against sustained QFT conversion was also observed using more stringent QFT thresholds^8^. Importantly, BCG showed protection against initial conversion at IFNγ >4.0IU/mL, which was associated with increased risk of TB disease in infants^30^, consistent with predictions from animal models^32^. 

A meta-analysis of observational studies of primary BCG vaccination reported a pooled estimate of 27% efficacy against initial *M.tb* infection and 71% efficacy against TB disease1^8^. Primary BCG vaccine efficacy against disease is highly variable in different populations, is greatest in mycobacteria-naïve individuals^33^and may last for 10 years^33,34^. Our findings suggest BCG revaccination of QFT-negative adolescents may provide additional benefit19. Two large cluster-randomized trials evaluated prevention of disease by BCG revaccination and did not demonstrate efficacy21,22. Neither trial enrolled based on *M.tb* or HIV infection status, or tested for prior mycobacterial sensitization or acquisition of *M.tb* infection. In Brazilian children aged 7-14 years, efficacy of BCG revaccination against TB disease was 9% after 5 years^21^ and 12% after 9 years, both estimates not statistically significant20. The trial was cluster-randomized, open-label, with no placebo, and the TB disease endpoint was determined from health service records^21^. However, a modest statistically significant efficacy signal (33%) was observed in children revaccinated at <11 years of age at one of two sites^20^. The second trial, a double-blind, randomized placebo-controlled trial of BCG revaccination among more than 46,000 people aged 3 months – 70 years showed no significant efficacy against confirmed TB disease (incidence rate ratio 1.43)^22^, in a Malawian community in which a trial of primary BCG vaccination had also shown no efficacy^35^. Based on our results and given the substantial differences in trial methodology, TB epidemiology and study populations, a trial of BCG revaccination for prevention of disease in *M.tb*-uninfected adolescents is justified in high TB incidence settings. Such a trial would also validate the POI strategy to de-risk TB vaccine development and allow identification of immune correlates of protection against disease. From a public health perspective, the potential risk of BCG disease in adolescents at high risk for HIV infection should be balanced against the potential benefits. 

A successful TB vaccine might function by several mechanisms, including prevention of initial *M.tb* infection, sustained infection, or progression to disease. Our results indicate that vaccination did not avert initial colonization, likely mediated by innate immunity, but allowed antigen trafficking to lymphoid tissues to trigger adaptive immunity (measured by initial QFT conversion). Rather, we hypothesize that vaccine-mediated QFT reversion is associated with enhanced bacterial control or clearance, likely mediated by collaborative adaptive and innate immune responses, which have been associated with sterilization of individual granulomas in non-human primates^36,37^. Although antigen-specific memory T cells measured by QFT can persist after bacterial clearance^22^, there is a positive correlation between *M.tb* replication in animal models and the magnitude of IFNγ responses to *M.tb*-specific antigens^25^. Indeed, transient TST conversion has been shown in humans and guinea pigs to be associated with reduced risk of TB disease compared with sustained conversion^12-14^. Further studies are required to understand clinical significance of QFT reversion and underlying immunological determinants. Comprehensive analyses are required to elucidate immune responses and mechanisms that correlate with protection, to guide new TB vaccine evaluation and design. 

Definitive interpretation of our findings is limited because there is no gold standard test for acquisition, persistence or clearance of *M.tb* infection. QFT has technical limitations, which we addressed by implementing optimized assay procedures^8^, utilizing alternative threshold definitions and serial testing. Testing only for initial QFT conversion in this trial would not have demonstrated efficacy; thus future POI trials should evaluate prevention of sustained conversion to avoid rejection of a potentially efficacious vaccine candidate. The POI trial design has potential to miss an impactful vaccine that prevents TB disease but not *M.tb* infection1^8^. Conversely, a vaccine that prevented sustained infection mainly in the ~90% of *M.tb*-infected individuals who naturally never develop disease would have little impact on TB prevention^4,5^. 

These findings confirm model predictions that vaccine efficacy against *M.tb* infection can be observed in a very high transmission setting^4^. It is unclear if our observations are generalizable to settings with lower *M.tb* transmission rates^21,22^. 

Our results raise important questions around the significance of prevention of *M.tb* infection for control of TB disease and provide a promising signal for BCG, other live mycobacterial and adjuvanted subunit vaccines. These encouraging findings provide impetus to re- evaluate BCG revaccination of *M.tb*-uninfected populations for prevention of disease^19^ and accelerate new TB vaccine development and illustrate the value of conducting human trials of TB vaccine candidates. 

## Supplementary Material

Supplementary Appendix
