# Supplementary material for: Prevention of Infection with Mycobacterium tuberculosis by H4:IC31® Vaccination or BCG Revaccination in Adolescents
Source: N Engl J Med. Author manuscript; Available in PMC 2018 Jul 12. (PMC5937161; doi:10.1056/NEJMoa1714021)
Supplement: Supplementary Appendix [file NEJMoa1714021_Hatherill_SuppApp.pdf]

Prevention of Infection with *Mycobacterium tuberculosis* by H4:IC31® Vaccination or BCG Revaccination in Adolescents:

Supplementary Appendix

Elisa Nemes, Ph.D, Hennie Geldenhuys, M.B.Ch.B., Virginie Rozot, Ph.D, Kathryn Tucker Rutkowski, M.Sc., Frances Ratangee, B.N., Nicole Bilek, Ph.D, Simbarashe Mabwe, M.Sc., Lebohang Makhethe, B.Sc., Mzwandile Erasmus, B.Sc., Asma Toefy, B.Sc., Humphrey Mulenga, M.P.H., Willem A. Hanekom, M.D., F.C.Paed., Steven G. Self, Ph.D, Linda-Gail Bekker, M.B.Ch.B., Ph.D, Robert Ryall, Ph.D, Sanjay Gurunathan, M.D., Carlos A. DiazGranados, M.D., Peter Andersen, D.V.M., D.M.Sc., Ingrid Kromann, B.Sc., Thomas Evans, M.D., Ruth D. Ellis, M.D., Bernard Landry, M.P.H., David A. Hokey, Ph.D, Robert Hopkins, M.D., F.A.C.P., Ann M. Ginsberg, M.D., Ph.D, Thomas J. Scriba, Ph.D, and Mark Hatherill, M.D., F.C.Paed., for the C-040-404 Study Team

C-040-404 Study Team members: Charmaine Abrahams, Marcelene Aderiye, Hadn Africa, Deidre Albertyn, Fadia Alexander, Julia Amsterdam, Denis Arendsen, Hanlie Bester, Elizabeth Beyers, Natasja Botes, Janelle Botes, Samentra Braaf, Roger Brooks, Yolundi Cloete, Alessandro Companie, Kristin Croucher, Ilse Davids, Guy de Bruyn, Bongani Diamond, Portia Dlakavu, Palesa Dolo, Sahlah Dubel, Cindy Elbring, Margareth Erasmus, Terence Esterhuizen, Christine Fattore, Sebastian Gelderbloem, Diann Gempies, Sandra Goliath, Peggy Gomes, Yolande Gregg, Elizabeth Hamilton, Johanna Hector, Roxanne Herling, Yulandi Herselman, Jane Hughes, Devin Hunt, Henry Issel, Helene Janosczyk, Lungisa Jaxa, Carolyn Jones, Jateel Kassiem, Sophie Keffers, Xoliswa Kelepu, Alana Keyser, Alexia Kieffer, Sandra Kruger, Maureen Lambrick, Phumzile Langata, Maria Lempicki, Marie-Christine Locas, Angelique Luabeya, Lauren Mactavie, Lydia Makunzi, Pamela Mangala, Clive Maqubela, Boitumelo Mosito, Angelique Mouton, Mariana Mullins, Julia Noble, Onke Nombida, Dawn O'Dee, Amy O'Neil, Rose Ockhuis, Saleha Omarjee, Fajwa Opperman, Dhaval Patel, Christel Petersen, Abraham Pretorius, Debbie Pretorius, Michael Raine, Rodney Raphela, Maigan Ratangee, Christian Rauner, Susan Rossouw, Surita Roux, Elisma Schoeman, Constance Schreuder, Cashwin September, Justin Shenje, Barbara Shepherd, Muki Shey, Heather Siefers, Eunice Sinandile, Danna Skea, Marcia Steyn, Jin Su, Sharon Sutton, Anne Swarts, Patrick Syntin, Michele Tameris, Petrus Tyambetyu, Arrie van der Merwe, Elize van der Riet, Dorothy van der Vendt, Denise van der Westhuizen, Anja van der Westhuizen, Elma van Rooyen, Ashley Veldsman, Helen Veltdsman, Emerencia Vermeulen, Sindile Wiseman Matiwane, Noncedo Xoyana.

TABLE OF CONTENTS

1 ADDITIONAL METHODS 3

1.1 PARTICIPANT RECRUITMENT 3

1.2 INCLUSION AND EXCLUSION CRITERIA 3

1.2.1 *Inclusion Criteria 3*

1.2.2 *Exclusion Criteria 4*

1.3 INTERVENTIONS 5

1.3.1 *H4:IC31 ..... 5*

1.3.2 *BCG 5*

1.3.3 *Placebo 6*

1.4 MEASUREMENTS 6

1.4.1 *QFT assay 6*

1.4.2 *PBMC Intracellular Cytokine Staining Assay 6*

1.5 STATISTICAL ANALYSES 8

1.5.1 *Sample size and interim analyses 8*

1.5.2 *Efficacy 9*

1.5.3 *Safety 11*

1.5.4 *PBMC ICS 11*

2 SUPPLEMENTARY FIGURES 12

FIGURE S1: IMMUNOGENICITY 12

FIGURE S2: QFT VALUES 13

3 SUPPLEMENTARY TABLES 14

TABLE S1: FLOW CYTOMETRY PANEL 14

TABLE S2: INCIDENCE OF ADVERSE EVENTS BY MEDDRA PREFERRED TERM 15

TABLE S3: EFFICACY ANALYSES WITH EXPLORATORY ENDPOINTS AND PER PROTOCOL STUDY POPULATION 18

4 SUPPLEMENTARY REFERENCES 20

1 ADDITIONAL METHODS

1.1 Participant recruitment

The protocol was approved by the Medicines Control Council of South Africa (Reference number: 20130826) and the Human Research Ethics Committee of the University of Cape Town (SATVI site reference: 471/2013; Emavundleni site reference: 615/2014). Participants were recruited from high schools or from the community at the South African Tuberculosis Vaccine Initiative (SATVI) Worcester research site and at the Emavundleni Research Centre in Nyanga, Western Cape Province of South Africa, where community members have historically been enthusiastically engaged in TB clinical research.

Individual participant assent was obtained after consent by the parent or legal guardian prior to screening.

1.2 Inclusion and exclusion criteria

1.2.1 Inclusion Criteria

Participants met all of the following criteria at the time of randomization:

1. Completed the written informed consent and assent process.
2. Had age ≥ 12 years and ≤ 17 years on Study Day 0 (D0).
3. Agreed to stay in contact with the study site for the duration of the study, provide updated contact information as necessary, and had no current plans to move from the study area for the duration of the study.
4. For female subjects: agreed to avoid pregnancy from 28 days prior to Study D0 and for the full duration of the study. Women physically capable of pregnancy (not sterilized and still menstruating or within 1 year of the last menses) in sexual relationships with men must use an acceptable method of avoiding pregnancy during this period. Acceptable methods of avoiding pregnancy include a sterile sexual partner, sexual abstinence (not engaging in sexual intercourse), hormonal contraceptives (oral, injection, transdermal patch, or implant), vaginal ring, intrauterine device (IUD), or the combination of a condom or diaphragm with spermicide.
5. Had general good health, confirmed by medical history and physical examination.
6. Had BCG vaccination at least 5 years ago documented through medical history or by presence of healed BCG scar.
7. Tested QFT negative at screening, using the manufacturer’s recommended threshold of ≥0.35IU/mL.

1.2.2 Exclusion Criteria

Participant had none of the following at the time of randomization:

1. Acute illness on Study D0.
2. Axillary temperature ≥37.5°C on Study D0.
3. Abnormal laboratory values from the most recent blood collected prior to randomization as follows:
   - Laboratory evidence of hematologic disease (white blood cell count <3000/mm^3 or >11,500/mm^3; hemoglobin <0.9 times the lower limit of normal of the testing laboratory, by age and gender; absolute neutrophil count <1300/mm^3; absolute lymphocyte count <1000/mm^3).
   - ALT, AST, alkaline phosphatase, total bilirubin, creatinine, blood urea nitrogen (BUN) >1.25 times the upper limit of normal of the testing laboratory.
4. Greater than Grade 1 on the Toxicity Scale urinalysis result (with the exception of hematuria in a menstruating female), or urinalysis abnormality judged clinically significant by the investigator.
5. History or evidence of any clinically significant systemic disease, or any acute or chronic illness that might affect the safety, immunogenicity, or efficacy of study vaccine in the opinion of the investigator.
6. History of treatment for active TB disease or latent *M.tb* infection.
7. History or evidence, including chest X-ray, of active TB disease.
8. Shared residence with an individual receiving anti-TB treatment, or known to have incompletely treated culture or smear positive TB, at screening.
9. History of autoimmune disease or immunosuppression.
10. Used immunosuppressive medication within 42 days before Study D0 (inhaled and topical corticosteroids are permitted).
11. Received immunoglobulin or blood products within 42 days before Study D0.
12. Received any investigational drug therapy or investigational vaccine within 182 days before Study D0, or planned participation in any other clinical trial during the study period.
13. Received investigational TB vaccine, other than BCG, at any time prior to Study D0.
14. Planned administration/administration of a licensed vaccine in the period starting 28 days before and ending 28 days after each dose of study vaccine.
15. History or laboratory evidence of any past or present possible immunodeficiency state including, but not limited to, any laboratory indication of HIV-1 infection.
16. History of allergic disease or reactions, including eczema, likely to be exacerbated by any component of the study vaccine.
17. History of alcohol or drug abuse.

18. All female subjects: currently pregnant or lactating/nursing; or positive urine pregnancy test during screening.
19. Received a tuberculin skin test (TST) within 3 months (90 days) prior to Study D0.
20. Any current medical, psychiatric, occupational, or substance abuse problems that, in the opinion of the investigator, will make it unlikely that the subject will comply with the protocol.

1.3 Interventions

1.3.1 H4:IC31

The current formulation of H4:IC31 is a field reconstituted vaccine with H4 polyprotein antigen and IC31 adjuvant supplied in different vials. The components are dissolved in a sterile aqueous buffer containing trishydroxymethylaminomethane (Tris) and sodium chloride (NaCl). The H4 antigen is a fusion protein created from two mycobacterial antigens, Ag85B and TB10.4. Ag85B is also referred to as α-antigen and is a 30-kDa mycolyl transferase protein[1,2]. TB10.4 is one of three members of the very similar ESAT-6 group of proteins found in *M.tb* culture supernatants. TB10.4 induces broad immune responses in T cells isolated from TB patients compared to BCG-vaccinated donors and unvaccinated donors[3,4].

The IC31 adjuvant is a proprietary adjuvant (Valneva, formerly Intercell, Vienna, Austria). IC31 is a combination of a leucine-rich peptide, named KLK, and a synthetic oligonucleotide, named ODN1a. KLK enhances the uptake of antigens into the antigen-presenting cell and increases the immune response to peptide antigens. ODN1a is a synthetic bacterial deoxyribonucleic acid (DNA) analogue that resembles a CpG pattern that will direct the adaptive immune response toward a T helper cell type-1 (Th1) pattern with production of interferon-γ (IFNγ) and interleukin-12 (IL12). The amount of adjuvant given may affect the immune response. The optimal molar ratio of KLK to ODN1a in mice is 25:1[5]. This same molar ratio is used in all formulations of H4:IC31 tested in clinical studies. For simplicity and clarity, amounts and concentrations of IC31 adjuvant are expressed as molar equivalents of KLK.
H4:IC31 was administered on D0 and D56 intramuscularly with a 22 gauge needle (0.5mL).

1.3.2 BCG

BCG Vaccine SSI was manufactured by Statens Serum Institut (SSI), Copenhagen, Denmark and imported and distributed in South Africa for the national immunization program by Biovac, Johannesburg, South Africa for the trial period. BCG Vaccine SSI is registered in

South Africa for prevention of TB in children and adults. The Statens Serum Institut in Copenhagen, Denmark derived this vaccine from the Danish BCG strain 1331. SSI BCG is supplied by the manufacturer in amber 10-dose vials containing 0.75 mg lyophilized SSI BCG. The vaccine was reconstituted with Sauton SSI diluent supplied by the manufacturer, according to the manufacturer's instructions. After reconstitution, 1 adult dose (0.1 mL) contains 2 to 8 x 10^5 CFU and was administered on D0 intradermally with a 22 gauge needle. Sauton SSI diluent is composed of magnesium sulphate, dipotassium phosphate, citric acid, monohydrate L-asparagine monohydrate, ferric ammonium citrate and glycerol at 85%, which is reconstituted with sterile water for injection.

1.3.3 Placebo

Saline volume equivalent to the H4:IC31 injection (0.5mL) was administered on D0 and D56 by intramuscular injection with a 22 gauge needle.

1.4 Measurements

1.4.1 QFT assay

The QFT assay was conducted according to the manufacturer's instructions, with implementation of more stringent parameters unless otherwise stated. Briefly, 1mL of blood was collected in each QFT tube and mixed by 10 manual inversions and 5 minutes on a tube rotator, incubated at 37°C within 2 hours from phlebotomy for 16 to 20 hours, and plasma was harvested without further delay. Supplementary quality control criteria, in addition to the manufacturer's algorithm, were implemented as described[6].

1.4.2 PBMC Intracellular Cytokine Staining Assay

Peripheral blood mononuclear cells (PBMC) were isolated from blood collected on D0 and D70 from participants in the safety and immunogenicity cohort. PBMCs were cryopreserved and shipped to Aeras, Cape Town, South Africa where a 13-colour intracellular cytokine staining (ICS) assay was performed[7]. PBMCs were thawed, suspended in R10 medium, and rested overnight. R10 medium is composed of 10% FBS (GemCell 100-500, triple filtered or equivalent), 55µM ßME (Gibco BRL 21985-023 or equivalent), 10 mM HEPES (Gibco BRL 15630-080 or equivalent), 2mM L-glutamine (Gibco BRL 25030-081 or equivalent), and 1 x penicillin-streptomycin (Gibco BRL 15140-122 or equivalent), in RPMI 1640 medium (Gibco/Invitrogen 11875-093 or equivalent). After overnight rest, cells were counted using

the Guava easyCyte Flow Cytometer; stimulation was only performed on samples with at least 70% viability.

Peptide stimulation was performed using dimethyl sulfoxide (DMSO, Sigma; negative control), phytohaemagglutinin (PHA, Remel; 1.0µg/mL; positive control), and peptide pools from Ag85B and TB10.4 (JPT, 1µg/peptide/mL; 15mers with an 11 amino acid overlap, pre-diluted in R10 medium containing CD107a-Alexa488, GolgiStop and GolgiPlug, 1 µL each [BD Biosciences, USA]). Stimulation (approximately 1 x 10^6 cells per condition) was for 6-7 hours at 37±3°C and 5±1% CO_2. BCG stimulation was performed using R10 medium (negative control), PHA (positive control), and BCG Vaccine (Statens Serum Institut, Denmark, reconstituted in R10 medium). PBMCs were stimulated at 37±3°C and 5±1% CO_2 with approximately 3 x 10^5 CFU/well of BCG Vaccine SSI diluted in R10 medium containing costimulatory antibodies CD28 and CD49d (BD Biosciences, USA; 1µL/well). After 2 hours CD107a-Alexa488, GolgiStop and GolgiPlug were added and the cells stimulated for an additional 6-7 hours at 37±3˚C and 5±1% CO_2.

After stimulation, prior to staining, the cells were washed with 1X PBS and stained with Aqua Live/Dead Fixable viability dye (Life Technologies, USA) before staining with fluorochrome-conjugated antibodies to surface markers CCR7, CD4, CD14, CD19 and CD45RO, then fixed and permeabilized (Cytofix/Cytoperm, BD Biosciences, USA) and stained for CD3, CD8, IFNγ, IL2, TNF, IL22, IL17, and CD154 (Supplementary table 1). Following incubation, cells were washed, fixed, and analyzed by flow cytometry. For ICS data acquisition, the BD LSR flow cytometer (BD Biosciences, US) was set to collect up to 150,000 viable CD3+ target cell events from each sample. All sample analysis was performed with FlowJo software (FlowJo LLC, USA) using the gating strategy illustrated by Graves *et al.*[7] and visualised using GraphPad Prism v7.0c.

Gating strategy, performed by an initial experienced operator, was identical within participants except for minor adjustments of individual gates. A second operator performed a quality control check and queries were discussed with the scientific supervisor. Data were exported and locked prior to unblinding and statistical analysis. Inclusion criteria for samples to be included in the immunogenicity were as follows: a) the difference between CD8 IFNγ+ cells in the negative control and CD8 IFNγ+ cells in the positive control should be ≥ 1% in at least one of the positive control samples; b) Number of acquired CD4+ and CD8+ events each ≥ 5,000; c) CD4+ population ≥ 10% of CD3+ population; d) CD8+ population ≥ 5% of CD3+ population.

1.5 Statistical analyses

1.5.1 Sample size and interim analyses

Sample size was based on detection of reduction in rate of *M.tb* infection as determined by QFT conversion. The trial was designed to distinguish QFT conversion rate reduction of 50% compared to placebo for each vaccine (H4:IC31 or BCG) with 80% power and a type 1 error rate of 10% (1-sided).

Design calculations were performed by computer simulation estimating the statistical operating characteristic of a conditional binomial test, which, due to relative rarity of the endpoint, provides a reliable approximation to the operating characteristic of the log-rank test. The following assumptions, based on preliminary data from the Worcester study site[8], were used in these simulations: i) Primary *M.tb* infection endpoint rate = 10% per year; ii) Rate of loss to follow-up = 7% per year; iii) Average rate of enrolment = 20 participants per week; iv) Maximum duration of follow-up to primary conversion = 24 months with 6-monthly study visits.

A total sample size of 990 (330 per study arm) was expected to provide 64 primary QFT conversion endpoints approximately 21 months after the first participant was enrolled.

Per protocol, follow-up leading to the primary analysis continued until at least 64 endpoints had accrued and a median follow-up duration of at least 15 months for individual participants had occurred. Following the primary analysis - at which point 77 participants had met the primary endpoint - the Independent Data Monitoring Committee (IDMC) recommended to extend to at least 24 months the follow-up of participants who converted QFT at Month 6 or 12, to assess the duration of sustained QFT conversion at EoS.

At the time of the primary analysis, members of the IDMC, along with members of the Joint Steering Committee (JSC) from Sanofi Pasteur and Aeras, were unblinded to study results at the by-group level. Members of the study team, who remained blinded until the end of the trial and after database lock, were involved in the development of the final Statistical Analysis Plan (SAP), which was finalized prior to database lock.

The final analyses associated with the primary and secondary endpoints remained entirely unchanged from the primary analysis. Some of the exploratory endpoints that had not been included within the primary analysis, including the evaluation of alternative QFT thresholds and the evaluation of sustained QFT conversions and late reversions at EoS, were proposed

by blinded members of the study team prior to database lock. While members of the JSC approved the final SAP, they were not involved in the selection of alternative QFT thresholds or the determination of methodology to evaluate trends in QFT over time.

The study was not powered to assess vaccine efficacy for prevention of TB disease. Based on studies in this population[8-10] we would expect to observe between 1 and 13 TB incident cases 95% of the time.

Unless otherwise indicated, SAS 9.4 (SAS Institute, Cary, NC) was used for data analysis, and the analyses were completed by Quintiles (now called IQVIA).

1.5.2 Efficacy

Prior to database lock and trial unblinding, we chose to prioritize the modified intent-to-treat analyses over the per-protocol analyses to be more reflective of a potential vaccine effect from real-world use rather than in a more idealistic clinical trial setting.

The trial's primary efficacy endpoint, time to first QFT conversion after D84 using the manufacturer's recommended threshold of IFNγ ≥ 0.35 IU/mL, was evaluated using a one-sided log-rank statistic to compare H4:IC31 versus placebo and BCG versus placebo without adjustment for multiplicity. The rationale for not adjusting for multiplicity to control the Type 1 error rate over the two tests is that the tests pertain to efficacy assessments of two unrelated vaccines. If these two evaluations were performed in two separate trials then no adjustment for multiplicity would be made. Thus, one should not be compelled to adjust for this multiplicity simply because the two evaluations are performed administratively in a single trial.

Estimates of vaccine efficacy were based on the hazard ratio estimate from a Cox regression model. Participants who did not experience a primary endpoint were censored at the date of their last available, non-positive QFT result; indeterminate results were treated as negative results. The log-rank statistic was evaluated using a one-sided test to reflect the way in which the trial was originally designed and powered, and two-sided 80% and 95% CI of vaccine efficacy are reported. Sensitivity analyses associated with the primary efficacy endpoint, including time to first QFT conversion after D0 (i.e., including conversions detected on D84) and time to event using interval-censoring, were also evaluated.

The secondary efficacy endpoint was defined as sustained QFT conversion without a change in QFT from positive to negative for six months after initial QFT conversion (i.e., QFT

≥ 0.35 IU/mL maintained at 3 and 6 months post-initial conversion). This endpoint was evaluated in the same manner as described above for the primary efficacy endpoint.

Several exploratory efficacy analyses further evaluated the vaccine ability to prevent or modulate infection:

- Median QFT levels at the time of initial conversion were compared between treatment groups using Mann-Whitney-Wilcoxon tests.
- Time to QFT reversion among those participants who converted was evaluated using a log-rank test.
- Time to first QFT conversion among those who ever reverted was evaluated using a log-rank test.
- Time to end-of-study sustained conversion (as opposed to sustained conversion for 6 months) was evaluated in the same manner as the secondary efficacy endpoint (described above).

The proportion of participants classified as converters according to alternative QFT thresholds (i.e., ≥0.2IU/mL; <0.2IU/mL at any time point prior to conversion and >0.7IU/mL at any time point post-D84; >0.7IU/mL; >4.0IU/mL) were compared across study arms. Vaccine efficacy was calculated based on the conditional binomial distribution, and 80% and 95% CI were calculated using the Clopper-Pearson method with mid-p correction.

Following an IDMC request at the time of the primary analysis to obtain Month 24 QFT results for all participants, including those who experienced conversions earlier in the study (at Month 6 or Month 12), additional exploratory analyses included an assessment of the QFT conversion and reversion rates at the end of the 24-month study follow-up period. Trends in QFT status were analysed similarly to the alternative QFT thresholds (i.e., based on a conditional binomial distribution and using the Clopper-Pearson method with mid-p correction for 80% and 95% CI) and were compared between treatment groups based on Pearson’s Chi-square for the following endpoints of interest:

- Participants who were QFT negative at Month 24 or the Month 24 call-back visit.
- Participants who never converted or who converted without sustained conversion.
- Participants who never converted or who converted with reversion and had non-positive 6-month post-initial-conversion QFT values.
- Participants who never converted or who converted with reversion and had non-positive end-of-study QFT values.

Note: Some analyses were based on subsets of participants determined by a post-randomization event such as reversion post-conversion, and some involved participants who experienced differential follow-up. These types of analyses have the potential to be biased, therefore they were not prioritized.

1.5.3 Safety

Safety outcomes included unsolicited adverse events (AEs; 28 days post each vaccination); systemic solicited AEs (7 days post each vaccination); injection site AEs (28 days post each vaccination for H4:IC31 and placebo; 84 days post vaccination for BCG); and AEs of special interest (AESIs; defined as events that were potentially immune mediated) and serious adverse events (SAEs), recorded at all available post-vaccination time points and for a minimum of 6 months after the last vaccination.

Post-vaccination laboratory and vital sign abnormalities were evaluated in the safety and immunogenicity cohort at each post-vaccination time point and overall and were included in the evaluation of AEs.

The incidence of any adverse event (AE), any solicited AE, any unsolicited AE, and any serious adverse event (SAE) were evaluated overall and by dose, relationship to vaccination, and highest severity. Adverse events were classified by MedDRA system organ class and preferred term.

1.5.4 PBMC ICS

For immunogenicity analyses, the median percentages of background-subtracted cytokine responses and changes from baseline by T cell type (CD4, CD8), antigen (Ag85B, TB10.4, BCG), and visit (D0, D70) were compared across study arms for responses including IFNγ and/or IL2 using Mann-Whitney-Wilcoxon tests. Median background-subtracted cytokine responses and their associated 95% CIs based on order statistics were also calculated for each treatment group by study day, T cell type, and antigen.

2 SUPPLEMENTARY FIGURES

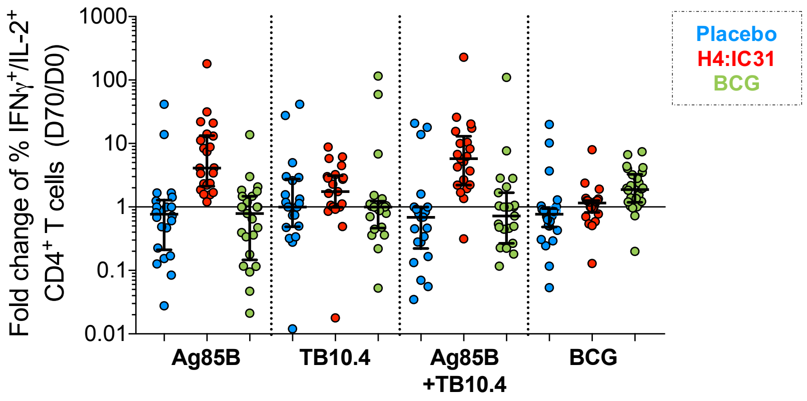

Figure S1: Immunogenicity

Vaccine-induced CD4 T cell responses measured by PBMC ICS. Ratio (fold change) of antigen-specific CD4 T cells expressing IFNγ and/or at D70 over D0 in each study arm (placebo = blue, H4:IC31 = red, BCG = green). Each dot represents an individual.

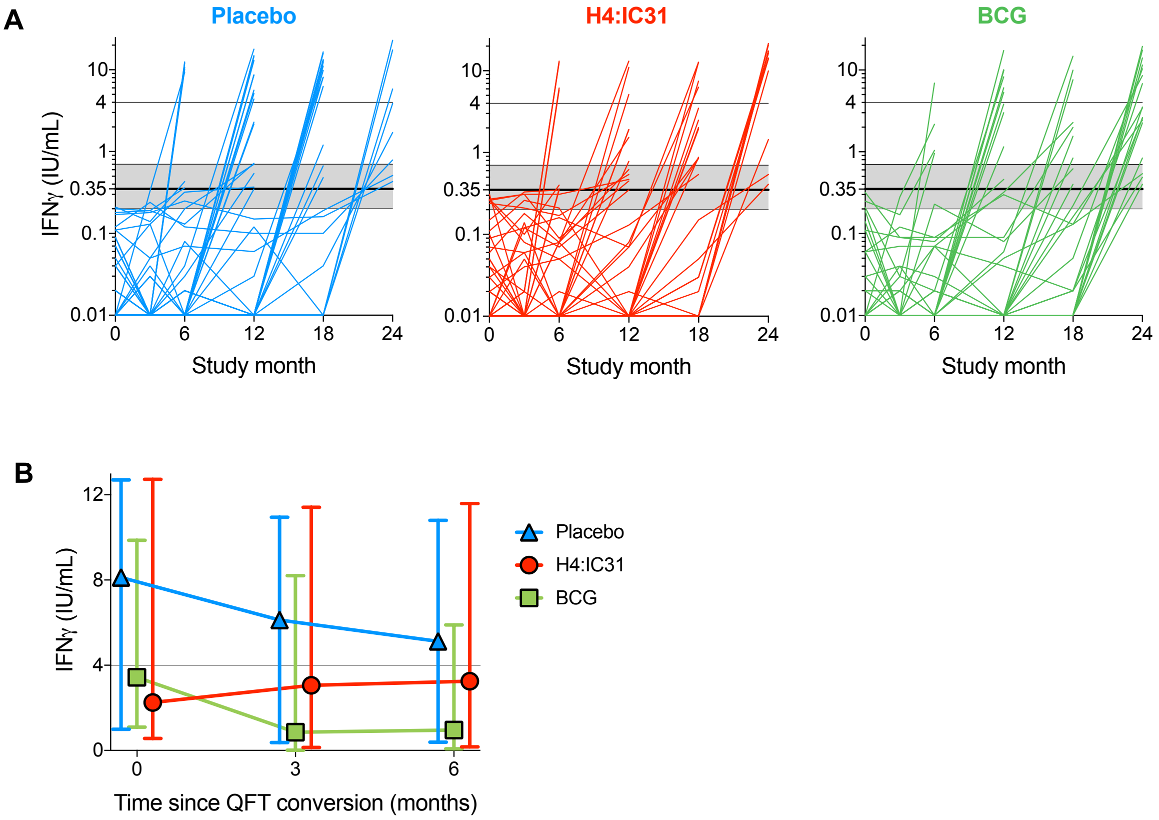

Figure S2: QFT values

(A) Longitudinal quantitative IFNγ values measured by QFT by study arm from time of enrolment in the study (Month 0) until initial QFT conversion (primary endpoint). Each line represents one individual; those who never converted are not shown. The solid line denotes the manufacturer's recommended threshold (0.35IU/mL); the shaded area denotes the uncertainty zone (0.2-0.7IU/mL); the horizontal line at 4.0IU/mL denotes an alternative QFT threshold applied in exploratory analyses. Values <0.01IU/mL were set to 0.01 to enable plotting on the log scale.

(B) QFT values in participants who met the primary endpoint at time of conversion and through six months post-conversion, including those who reverted. Medians and interquartile ranges are shown. The horizontal line at 4.0IU/mL denotes an alternative QFT threshold applied in exploratory analyses.

3 SUPPLEMENTARY TABLES

Table S1: Flow cytometry panel

Antibody	Fluorochrome	Clone	Supplier	Rationale
CD3	ECD	UCHT1	Beckman Coulter	Lineage
CD4	APC-eFluor780	RPA-T4	eBioscience	Lineage
CD8	AF700	HIT8a	BioLegend	Lineage
CD45RO	BV785	UCHL1	BioLegend	Memory
CCR7	BV605	G043H7	BioLegend	Memory
IL2	PE	MQ1-17H12	BD	Th1
IFNγ	V450	B27	BD	Th1
TNF	PE-Cy7	MAb11	BD	Th1
IL17A	PerCP-Cy5.5	BL168	BioLegend	Th17
IL22	APC	IL22JOP	eBioscience	Th22
CD154	PE-Cy5	TRAP1	BD	Help
CD107a	AF488	H4A3	BioLegend	Cytotoxicity
CD14	V500	M5E2	BD	Dump
CD19	V500	HIB19	BD	
Live/Dead	Aqua	L34957	Life Technologies	

Table S2: Incidence of adverse events by MedDRA preferred term

	Placebo (N=329)		H4:IC31 (N=330)		BCG (N=330)		Total (N=989)
Variable	n (%)	95% CI	n (%)	95% CI	n (%)	95% CI	n (%)
**AE Categories**							
Any AE	104 (31.6)	26.8 – 36.8	120 (36.4)	31.3 – 41.7	326 (98.8)	97.1 – 99.6	550 (55.6)
Mild AE	81 (24.6)		105 (31.8)		307 (93.0)		493 (49.8)
Moderate AE	21 (6.4)		14 (4.2)		18 (5.5)		53 (5.4)
Severe AE	2 (0.6)	0.1 – 2	1 (0.3)	0.0 – 1.5	1 (0.3)	0.0 – 1.5	4 (0.4)
Severe, related AE	0	0.0 – 0.9	0	0.0 – 0.9	0	0.0 – 0.9	0
Solicited AE	48 (14.6)	11.1 – 18.7	72 (21.8)	17.6 – 26.5	294 (89.1)	85.4 – 92.1	414 (41.9)
Unsolicted AE (non-serious)	80 (24.3)	19.9 – 29.2	80 (24.2)	19.8 – 29.1	288 (87.3)	83.3 – 90.6	448 (45.3)
Local solicited AE	25 (7.6)	5.1 – 10.9	44 (13.3)	10.0 – 17.3	291 (88.2)	84.4 – 91.3	360 (36.4)
Systemic solicited AE	38 (11.6)	8.4 – 15.4	45 (13.6)	10.2 – 17.7	30 (9.1)	6.3 – 12.6	113 (11.4)
[a]SAE	7 (2.1)	0.9 – 4.2	5 (1.5)	0.6 – 3.3	7 (2.1)	0.9 – 4.2	19 (1.9)
SAE (death)	1 (0.3)	0.0 – 1.5	0	0.0 – 0.9	0	0.0 – 0.9	1 (0.1)
AESI	0	0.0 – 0.9	0	0.0 – 0.9	0	0.0 – 0.9	0
**Local And Regional AEs**							
Injection site Pain	20 (6.1)		34 (10.3)		45 (13.6)		99 (10.0)
Injection site Swelling	8 (2.4)		15 (4.5)		172 (52.1)		195 (19.7)
Injection site Induration	1 (0.3)		1 (0.3)		166 (50.3)		168 (17.0)
Injection site Erythema	6 (1.8)		8 (2.4)		147 (44.5)		161 (16.3)

Injection site Ulcer	0		0		104 (31.5)		104 (10.5)
Injection site Scar	0		0		45 (13.6)		45 (4.6)
**Systemic AEs**							
Lymphadenopathy	0		1 (0.3)		2 (0.6)		3 (0.3)
Headache	23 (7.0)		24 (7.3)		23 (7.0)		70 (7.1)
Fatigue	18 (5.5)		16 (4.8)		10 (3.0)		44 (4.4)
Myalgia	11 (3.3)		17 (5.2)		7 (2.1)		35 (3.5)
Arthralgia	6 (1.8)		5 (1.5)		5 (1.5)		16 (1.6)
Pyrexia	1 (0.3)		3 (0.9)		2 (0.6)		6 (0.6)
Chills	5 (1.5)		6 (1.8)		3 (0.9)		14 (1.4)
Nausea	3 (0.9)		12 (3.6)		10 (3.0)		25 (2.5)
Diarrhoea	5 (1.5)		3 (0.9)		6 (1.8)		14 (1.4)
Upper respiratory tract infection	26 (7.9)		31 (9.4)		7 (2.1)*		64 (6.5)
Blood pressure diastolic increased	4 (1.2)		0		2 (0.6)		6 (0.6)
Blood pressure systolic increased	0		6 (1.8)		0		6 (0.6)
Platelet count increased	2 (0.6)		3 (0.9)		2 (0.6)		7 (0.7)
White blood cell count increased	0		5 (1.5)		2 (0.6)		7 (0.7)
Neutrophil count decreased	3 (0.9)		1 (0.3)		2 (0.6)		6 (0.6)
Neutrophil count increased	0		2 (0.6)		2 (0.6)		4 (0.4)
Haemoglobin decreased	3 (0.9)		2 (0.6)		0		5 (0.5)

AE: Adverse event; SAE: Serious Adverse Event; BCG: Bacillus Calmette-Guerin; CI: Confidence interval; n = number of participants who experienced at least one AE in each category (participants with multiple events in a category are counted only once in that category); N = total

number of participants in the safety analysis set in each treatment group; % = percentage of participants in each category relative to the total number of participants in the safety analysis set in each treatment group. [a] SAE (listed by preferred term) included breast abscess (n=1); cellulitis (n=1); subcutaneous abscess (n=1); near drowning (n=1); completed suicide (n=1); eclampsia (n=1) and premature rupture of membranes (n=1) in the Placebo arm (subtotal n=7); pyelonephritis (n=2); viral meningitis (n=1); road traffic accident (n=1) and intentional self-injury (n=1) in the H4:IC31 arm (subtotal n=5); and gastroenteritis (n=2); chest injury (n=1); thermal burn (n=1); intentional self-injury (n=1); suicide attempt (n=1) and small intestinal obstruction (n=1) in the BCG arm (subtotal n=7) (total n=19). *p=0.0003 calculated by two by three Chi Square test.

Table S3: Efficacy analyses with exploratory endpoints and per protocol study population

Arms		Placebo	H4:IC31					BCG				
				Vaccine vs. Placebo					Vaccine vs. Placebo			
Endpoint	Quantiferon conversion threshold	n/N (%)	n/N (%)	Point Est (%)	80% CI	95% CI	p-val	n/N (%)	Point Est (%)	80% CI	95% CI	p-val
**mITT Exploratory endpoints**												
Never QFT converted or converted without sustained conversion[1]	≥ 0.35IU/mL	270/307 (87.9)	279/305 (91.5)	**30.3**[2]	3.2, 50.0	-15.0, 58.2	0.15[3]	289/311 (92.9)	**43.3**[2]	20.0, 60.0	4.3, 67.0	0.04[3]
QFT conversion[4]	≥ 0.2IU/mL	51/296 (17.2)	52/295 (17.6)	**-1.8**[5]	-31.2, 21.0	-50.1, 31.0	1.0[6]	47/300 (15.7)	**12.3**[5]	-13.7, 32.4	-30.5, 41.2	0.74[6]
QFT conversion[7]	< 0.2 to > 0.7IU/mL	41/310 (13.2)	34/308 (11.0)	**18.5**[5]	-9.9, 39.6	-28.6, 48.6	0.47[6]	36/312 (11.5)	**15.0**[5]	-14.0, 36.7	-33.2, 46.0	0.63[6]
**Per Protocol Primary and Secondary endpoints**												
QFT conversion[8]	≥ 0.35IU/mL	49/306 (16)	39/297 (13.1)	**18.7**[2]	-7.1, 38.2	-23.9, 46.6	0.17[3]	41/312 (13.1)	**20.8**[2]	-3.9, 39.6	-20.0, 47.7	0.13[3]
Sustained QFT conversion[9]	≥ 0.35IU/mL	36/306 (11.8)	24/297 (8.1)	**32.1**[2]	4.7, 51.5	-13.9, 59.5	0.07[3]	21/312 (6.7)	**45.8**[2]	22.9, 61.9	7.1, 68.4	0.01[3]

**Per Protocol Exploratory endpoint**												
Sustained QFT conversion[10]	<0.2 to >0.7IU/mL	31/306 (10.1)	24/297 (8.1)	**21.5**[2]	-11.3, 44.6	-33.8, 53.9	0.19[3]	19/312 (6.1)	**42.1**[2]	15.9, 60.2	-2.5, 67.3	0.03[3]
**Randomized analysis set**												
QFT conversion using all Post-Day 0 conversions[11]	≥ 0.35 U/mL	67/329 (20.4)	63/331 (19.0)	**6.0**[2]	-17.7, 24.9	-32.6, 33.4[3]	0.75	57/330 (17.3)	**16.7**[2]	-4.9, 33.9	-18.5, 41.5	0.15[3]

[1] Denominators are based on the number of participants who did not have two missing QFT values within 6 months post-initial conversion.

[2] Vaccine efficacy point estimates and 80% CI and 95% CI are based on the hazard ratio estimated from the Cox regression model (two-sided).

[3] P-values are based on a one-sided log-rank test compared to placebo. No multiplicity adjustment was done for p-values.

[4] QFT conversion from negative (< 0.2 IU/mL) at Baseline and Day 84 to positive (≥ 0.2 IU/mL) at any time point through end of study.

[5] Vaccine efficacy point estimates and 95% CI are calculated based on the conditional binomial (Clopper-Pearson method with mid-p correction).

[6] Two-sided p-values are based on Pearson Chi-square test.

[7] QFT conversion from negative (< 0.2 IU/mL) at any time point prior to conversion and positive (> 0.7 IU/mL) at any time point through end of study.

[8] QFT conversion from negative (< 0.35 IU/mL) at Day 84 to positive (≥ 0.35 IU/mL) at any time point through end of study in the Per Protocol population.

[9] QFT conversion from negative (< 0.35 IU/mL) at Day 84 to positive (≥ 0.35 IU/mL) at any time point through end of study, and without a change in QFT from positive to negative through 6 months after QFT conversion in the Per Protocol population.

[10] QFT conversion from negative at Day 84 to positive at any time point through end of study, using an alternative threshold of < 0.2 IU/mL at any time point prior to conversion and > 0.7 IU/mL at conversion and maintained through 6 months after initial conversion, in the Per Protocol population.

[11] QFT conversion from negative (< 0.35 IU/mL) at Baseline to positive (≥ 0.35 IU/mL) at any time point through end of study in the Randomized analysis population.

4 SUPPLEMENTARY REFERENCES

1. Horwitz MA, Harth G, Dillon BJ, Maslesa-Galic S. Recombinant Bacillus Calmette-Guerin (BCG) vaccines expressing the *Mycobacterium tuberculosis* 30-kDa major secretory protein induce greater protective immunity against tuberculosis than conventional BCG vaccines in a highly susceptible animal model. Proc Natl Acad Sci U S A 2000;97:13853-8.
2. Belisle JT, Vissa VD, Sievert T, Takayama K, Brennan PJ, Besra GS. Role of the major antigen of *Mycobacterium tuberculosis* in cell wall biogenesis. Science 1997;276:1420-2.
3. Skjot RL, Oettinger T, Rosenkrands I, et al. Comparative evaluation of low-molecular-mass proteins from *Mycobacterium tuberculosis* identifies members of the ESAT-6 family as immunodominant T-cell antigens. Infect Immun 2000;68:214-20.
4. Skjot RL, Brock I, Arend SM, et al. Epitope mapping of the immunodominant antigen TB10.4 and the two homologous proteins TB10.3 and TB12.9, which constitute a subfamily of the ESAT-6 gene family. Infect Immun 2002;70:5446-53.
5. Lingnau K, Egyed A, Schellack C, Mattner F, Buschle M, Schmidt W. Poly-L-arginine synergizes with oligodeoxynucleotides containing CpG-motifs (CpG-ODN) for enhanced and prolonged immune responses and prevents the CpG-ODN-induced systemic release of pro-inflammatory cytokines. Vaccine 2002;20:3498-508.
6. Nemes E, Rozot V, Geldenhuys H, et al. Optimization and interpretation of serial QuantiFERON testing to measure acquisition of *Mycobacterium tuberculosis* Infection. Am J Respir Crit Care Med 2017;196:638-48.
7. Graves AJ, Padilla MG, Hokey DA. OMIP-022: Comprehensive assessment of antigen-specific human T-cell functionality and memory. Cytometry A 2014;85:576-9.
8. Mahomed H, Hawkridge T, Verver S, et al. The tuberculin skin test versus QuantiFERON TB Gold in predicting tuberculosis disease in an adolescent cohort study in South Africa. PLoS One 2011;6:e17984.
9. Machingaidze S, Verver S, Mulenga H, et al. Predictive value of recent QuantiFERON conversion for tuberculosis disease in adolescents. Am J Respir Crit Care Med 2012;186:1051-6.
10. Andrews JR, Hatherill M, Mahomed H, et al. The dynamics of QuantiFERON-TB gold in-tube conversion and reversion in a cohort of South African adolescents. Am J Respir Crit Care Med 2015;191:584-91.
